# Preparation and extraction of chorion proteins from *Salmo salar* embryos at the pigmented eye stage for electrophoresis with SDS-polyacrylamide gel

**DOI:** 10.1016/j.mex.2023.102533

**Published:** 2023-12-23

**Authors:** Maritza Pérez-Atehortúa, Stefania E. Short, Cristian Aranzaez-Rios, Jorge Farías, Ricardo Pinheiro S. Oliveira, Wellison Amorim Pereira, Jennie Risopatrón, Iván Valdebenito, Elías Figueroa Villalobos

**Affiliations:** a Doctoral student in Agricultural Sciences, Faculty of Natural Resources, Catholic University of Temuco, Temuco 4781312 Chile; b Nucleus of Research in Food Production, Faculty of Natural Resources, Catholic University of Temuco, Temuco 4781312 Chile; c Department of Chemical Engineering, Faculty of Engineering and Sciences, La Frontera University, Temuco, Chile; d Laboratory of Microbial Biomolecules, School of Pharmaceutical Sciences, University of São Paulo, Rua Do Lago, 250, Cidade Universitária, São Paulo 05508 000, Brazil; e Center of Excellence in Biotechnology on Reproduction (BIOREN-CEBIOR), Faculty of Medicine, La Frontera University, Temuco 4811230, Chile

**Keywords:** Atlantic salmon, Egg envelope, Egg shell, Fish, Teleost, SDS-page, Method of preparation and extraction of chorion proteins from embryos at 280 accumulated thermal units (ATU) of Atlantic salmon (*Salmo salar*) for protein characterization by SDS-PAGE electrophoresis

## Abstract

The chorion fulfills important functions in fish embryos, including protecting the embryo during development. The characterization of the protein profile of this envelope could be used as a bioindicator in the evaluation of the quality of embryonic development. The object of this work was to validate a standardized protocol for protein extraction from chorion of *Salmo salar* embryos at 280 accumulated thermal units (ATU) by comparing and combining existing methods. The protocol consists of consecutive washing of the chorion samples followed by protein extraction with the solution that was named SDS solution (Tris–HCl 100 mM (pH 8), Urea 8 M, 1% SDS, β-mercaptoethanol 300 mM and EGTA 10 Mm, and 1% protease inhibitor cocktail) and mechanical methods. Protein extraction is enhanced by a working temperature of 75 °C and use of a disperser. The protein concentration was quantified by Bradford Assay. After extraction, the samples were diluted (dilution factor 10) before reading against the calibration curve. After gel electrophoresis with a load of 3 µg of protein, staining showed more than 4 bands, with molecular weights between 25 kDa and 180 kDa.

• The protein profile of fish chorion was between 25 kDa and 180 kDa.
• Solution containing 1% SDS allows a higher extraction of proteins from the chorion of Atlantic salmon embryos with 280 ATU.
• Chorion protein identification is a valuable tool in determining gamete and embryo quality in fish.

## Graphical abstract

**Figure:**
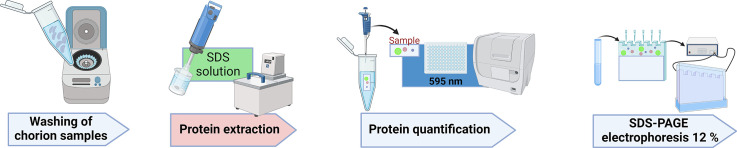

Specifications table

**Table:** 

Subject area:	Agricultural and Biological Sciences
More specific subject area:	Fish egg quality, Fish reproduction
Name of your method:	Method of preparation and extraction of chorion proteins from embryos at 280 accumulated thermal units (ATU) of Atlantic salmon (*Salmo salar*) for protein characterization by SDS-PAGE electrophoresis
Name and reference of original method:	The methodology was adapted according to the references in Supplementary Table 1, highlighting the following: Modig et al. (2008) Analysis of vitelline envelope synthesis and composition during early oocyte development in gilthead seabream (Sparus aurata). Mol Reprod Dev 75:1351–1360. 10.1002/mrd.20876 Oppen-Berntsen et al. (1990). The major structural proteins of cod (*Gadus morhua*) eggshells and protein crosslinking during teleost egg hardening. Developmental Biology, 137(2), 258–265. 10.1016/0012–1606(90)90,252-E
Resource availability:	NA

## Method details

### Background

Choriogenins (Chg) are a type of glycoproteins which make up the chorion, an acellular envelope that surrounds the oocytes of fish. Choriogenins fulfil multiple functions, including protecting the embryo against physical-chemical factors in the incubation environment until hatching [1], [2], [3], [4], [5], [6]. Depending on the species and/or stage of development, this envelope may be made up of two or more glycoprotein subunits [4,^7^,8]. For example, four glycoprotein subunits with molecular weights of 107 kDa, 92 kDa, 38 kDa and 31 kDa have been reported for Atlantic salmon (*Salmo salar*), identified in normal chorion of post-hatched embryos [9]. Four subunits have likewise been identified in the ovulated eggs of rainbow trout (*Oncorhycus mykiss*), with weights of 129 kDa, 62 kDa, 54 kDa and 47 kDa [10].

**Fig. 1:**
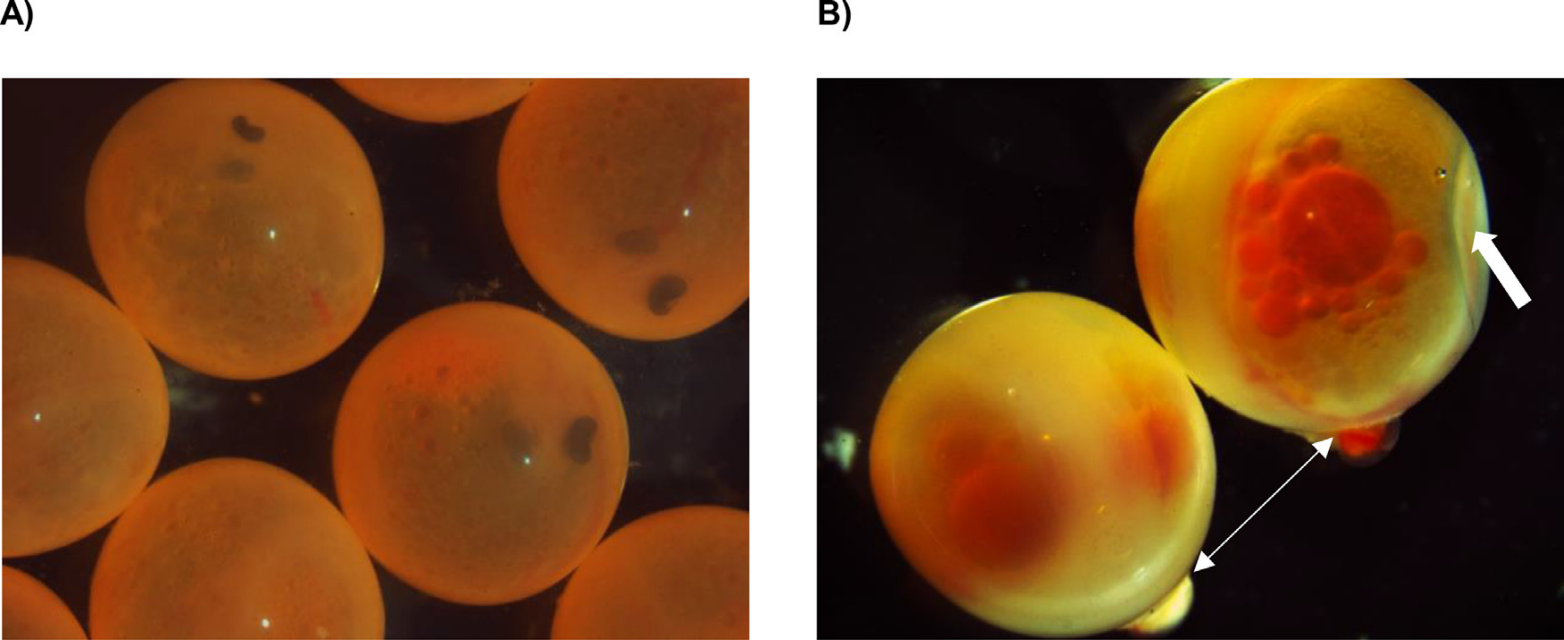
(A) Normal chorion and, (B) with presence of perforations (double arrow) and soft (thick arrow), of Atlantic salmon (*Salmo salar*) embryos at the pigmented eye stage (280 ATU).

Some malformations have been reported for salmonids, such as soft chorion (Fig. 1), hard chorion, and chorion with perforations and/or with disc, leading to embryonic losses [5,9]. Hard chorion is a malformation that results in embryo death, as the embryos cannot break through the chorion at hatching. According to Jaramillo et al. [9], hard chorion in *S. salar* was associated with increased molecular weights compared to normal chorion, with values of 179 kDa, 157 kDa, 64 kDa and 55 kDa. Although the molecular weight of different choriogenins has been identified by means of SDS-PAGE in different fish species, including *S. salar*, for protein extraction, the mechanical techniques (sonicator, porcelain mortar, liquid nitrogen, homogenizer), and/or the chemical techniques (e.g. concentration of the reagents, combination of different reagents such as SDS, β-mercaptoethanol, etc.), are not entirely clear, making replication difficult. In the present work, several techniques for chorion washing and protein extraction from *S. salar* embryos at the pigmented eye stage (280 ATU) were tested in order to standardize a protocol to be used for the preparation of samples for SDS-PAGE electrophoresis, and subsequent analysis of the specific subunits of the protein. This methodology was initially performed with the normal chorion, i.e. without any alteration (Fig. 1), as a starting point for a later identification of the protein profile of the cases associated with the different malformations. The treatment methods chosen for validation are summarized in Table 1.

**Table 1:** Treatments for chorion protein extractions.

Validation Phase 1			
Chorion washing	Protein extraction		Precipitation
	Chemical	Mechanical	
Liquid nitrogen	SDS	Sonicator	Y
	SDS	Homogenizer	Y
	SDS	Mortar	Y
	SDS	Liquid nitrogen	Y
	BL	Sonicator	N
	BL	Homogenizer	N
	BL	Mortar	N
	BL	Liquid nitrogen	N
Without liquid nitrogen	SDS	Sonicator	Y
	SDS	Homogenizer	Y
	SDS	Mortar	Y
	SDS	Liquid nitrogen	Y
	BL	Sonicator	N
	BL	Homogenizer	N
	BL	Mortar	N
	BL	Liquid nitrogen	N
**Validation Phase 2**			
Without liquid nitrogen	1% SDS	Mortar	Y
	BL		N
**Validation** Phase 3			
Without liquid nitrogen	SDS	Homogenizer (Digital Disperser)	Y
Without liquid nitrogen	BL		N
Without liquid nitrogen	U8M		N
**Final validation phase**			
Without liquid nitrogen	SDS	Homogenizer (Digital Disperser)	Y*

## Method details

### Required reagents

• EDTA
• NaCl
• Protease inhibitor cocktail (abcam ab271306)
• Tris/HCl
• Urea
• Sodium Dodecyl Sulfate (SDS)
• β-mercaptoethanol
• EGTA
• Ammonium persulfate (APS)
• Acrylamide/Bis Acrylamide
• Ethanol
• Isopropanol
• Glycerol
• Distilled water (H_2_Od)
• Acetone
• Sucrose
• Medium Potato Dextrose Agar Acid (PDA)
• Medium Luria–Bertani (LB)
• Methanol
• Isopropanol
• Dye Reagent (*Bradford*, BIO-RAD Cat #500–0006)
• Brilliant Coomasie Blue R-250 staining (VWR® LIFE SCIENCE CAS 6104–59–2)
• Bovine serum albumin (BSA)
• Bromophenol blue
• Tween-20
• Phenylmethylsulfonyl fluoride (PMSF)
• Pre-stained Protein Ladder 240 kDa Maestrogen (Cat # MG-02,102–250)

### Required equipaments

• Electrophoretic chamber (Mini-PROTEAN® Tetra Handcast Systems)
• Refrigerated centrifuge. For this protocol, the Eppendorf Centrifuge 5804 R was used
• Micropipettes
• Micropipette tips
• Eppendorf microcentrifuge tubes
• Falcon tubes
• Perkin Elmer LLC Pico Glass Vial, 7 mL
• Spectrophotometer multimodal microplates (SynergyTM HT)
• Erlenmeyer, measuring cylinders and beakers for the preparation of solutions
• Spatulas
• Analog 2 Block Heater (VWR® Cat #12,621–110)
• MiniSpin (Eppendorf® MiniSpin® AG 22,331)
• Rocker Shaker (Labnet Cat #1–72,025)
• Homogenizer (IKA™ ULTRA-TURRAX™ T 18 Digital Disperser)

### Biological material

Chorion samples were obtained from embryos of Atlantic salmon (*Salmo salar*) at the eye-pigmented stage (280 Accumulated thermal units - ATU). The study was carried out in accordance with the regulations of the Research Ethics Commission of the Universidad Católica de Temuco, Chile, with document number 112,201/22.

### General considerations of the methodology

Within the process of choosing and methodology standardizing the several points were considered:

• Given that the chorion is exposed to the incubator environment, samples may be contaminated with bacteria, fungi and other particles present in the water. The samples were first washed with Solutions 1 and 2 (Table 2; proposed respectively by Oppen-Berntsen et al. [15] and Modig et al. (2008)). Between these two washes, some of the samples were macerated in liquid nitrogen to further reduce the possible presence of bacteria or fungi, while others were not; apart from avoiding chorion activation, as indicated by the authors, it was observed that this facilitates the elimination of excess yolk from Atlantic salmon embryos. After washing with each of the two solutions, with and without liquid nitrogen treatment, samples of the supernatant and the chorion were taken to perform a fungal culture (in PDA medium) and a bacteria culture (in LB medium); the cultures were observed daily for seven days, to confirm the absence of these microorganisms. No population growth was observed after either wash, with or without liquid nitrogen treatment (Fig. S1). Moreover, when liquid nitrogen was used, initial signs of sample loss were observed; the use of liquid nitrogen between washes was therefore discarded.

**Table 2:** Summary of the solutions used (with their chemical composition) during the different stages of washing, extraction and quantification of the chorion proteins from Atlantic salmon (*Salmo salar*) embryos, and their subsequent electrophoresis with SDS-PAGE and gel staining.

Reagents	Solutions composition
**Washing of chorion samples**	
Wash solution 1 (pH 8.5)	EDTA 100 mM, NaCl 500 mM and protease inhibitor 1 µL/100 mL
Wash solution 2 (pH 8.5)	NaCl 500 mM and protease inhibitor 1 µL/100 mL
**Extraction of chorion proteins**	
Extraction solution urea 8 M contend SDS1% (SDS)	Tris–HCl 100 mM (pH 8), Urea 8 M, 1% SDS, β-mercaptoethanol 300 mM and EGTA 10 Mm and 1% protease inhibitor cocktail
Extraction solution buffer lysis (BL)	Tris 50 mM, NaCl 100 mM, EDTA 1 mM, EGTA 2.5 mM, Tween-20 0.1% and PMSF 100 µg/mL
Extraction solution urea 8 M (U8M)	Urea 8 M and 1% protease inhibitor cocktail
**Gels and running buffer**	
Separating gel 12% (10 mL)*	Buffer lower (2.5 mL), A/BA (4.08 mL), H_2_Od (3.25 mL), SDS 10% (100 µL), APS (150 µL), TEMED (20 µL)
Concentrating gel 4% (5 mL)*	Buffer upper (1.25 mL), A/BA (0.65 mL), H_2_Od (3.05 mL), SDS 10% (50 µL), APS (60 µL), TEMED (20 µL)
Running buffer 5X (pH 8.3)	Tris 0.125 M, Glycine 0.96 M, SDS 0.1% (stock solution)
Buffer lower	Tris 1.5 M (pH 8.8)
Buffer upper	Tris 0.5 M (pH 6.8)
Blue Loading Buffer (1X)	62.5 mM Tris-HCl (pH 6.8), 2% (w/v) SDS, 10% glycerol, Bromophenol blue 0.01%0.01% (w/v)
**Gels staining**	
Brilliant Coomasie Blue staining	1 g/L Brillian Coomasie Blue, 10% acetic acid, 40% methanol
Distaining solution	10% acetic acid, 40%methanol

⁎ To prepare two gels.
• Several extraction methodologies were found (Table S1) and combinations of these (Phase 1, Table 1) were tested by measuring the protein concentration obtained with each. The two techniques chosen for validation in Phase 2 were those that presented the best ratio between the protein concentration obtained (µg/mL) and the weight of the sample used for the extraction (µg), as shown in Table S2. Considering that the reagents contained in the SDS extraction solution (1% SDS, Urea 8 M, β-mercaptoethanol, see Table 2) present interference with colorimetric methods for protein quantification [11], [12], [13], and that with the Bradford method the reagent with the greatest interference was 1% SDS, two alternatives were tested. Protein extraction from the chorion using BL solution and without precipitation (Table 2) was used for comparison, as the concentration of its reagents did not interfere with Bradford; the proteins extracted with the SDS solution were precipitated as explained below [11].

SDS: SDS solution (Tris HCl 100 mM, Urea 8 M, 1% SDS, β-mercaptoethanol 300 mM and EGTA 10 Mm and 1% protease inhibitor cocktail); BL: Buffer lysis protein extraction solution (Tris 50 mM, NaCl 100 mM, EDTA 1 mM, EGTA 2.5 mM, Tween-20 0.1% and PMSF 100 µg/mL); U8M: Urea 8 M solution (Urea 8 M and 1% protease inhibitor cocktail). Precipitation was only applied to treatments in which SDS solution was used, due to interference of some components of the solution with the Bradford method of protein concentration quantification. Y: protein precipitation was performed; N: protein precipitation was not performed. * Dilution of proteins after extraction (dilution factor 10).

**Fig. 2:**
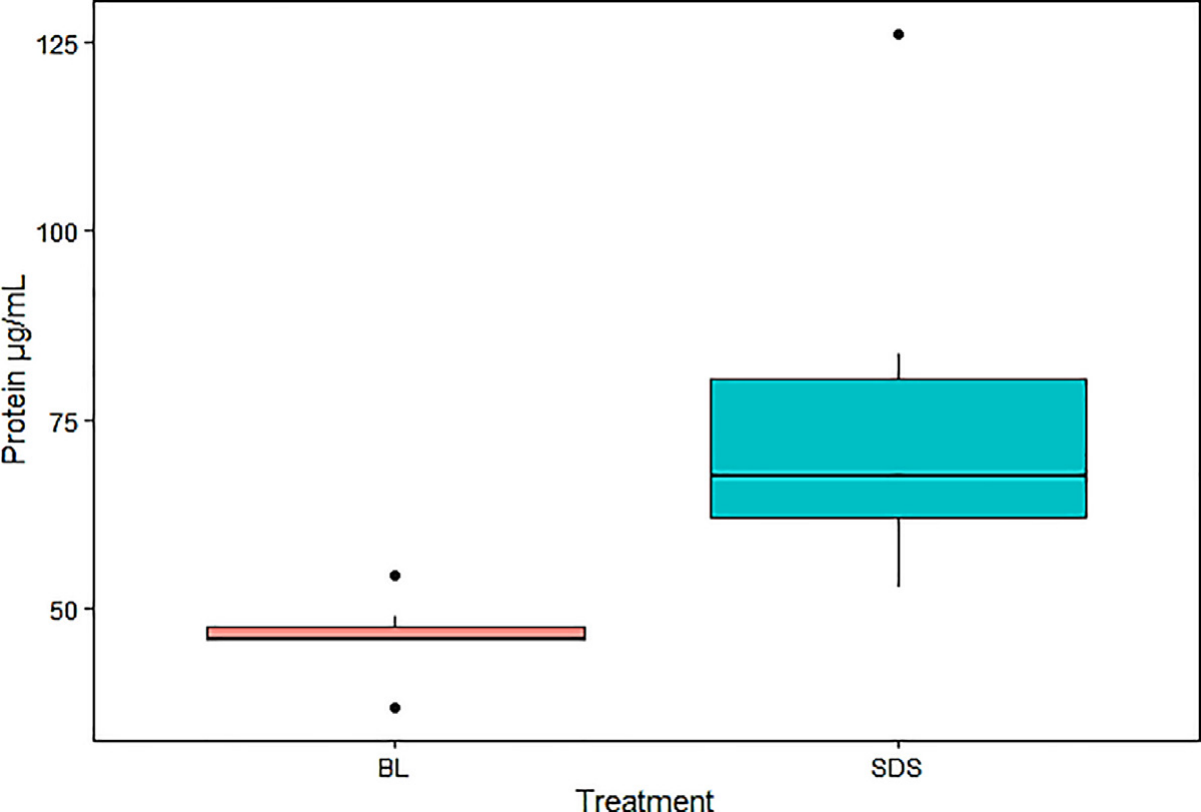
Protein concentration obtained in Phase 2 of the methodology for protein extraction from the chorion of Atlantic salmon (*Salmo salar*) embryos at the pigmented eye stage (280 ATU). With a significance level of 95%, a significant difference (*p* = 0.013) was found between the SDS (76.44 ± 26.39 µg/mL) and Buffer Lysis (BL) (46.31 ± 5.16 µg/mL) treatments, both using a mortar as the mechanical technique. t-value = 2.9771; the size of effect measured by Cohen's d was large (1.656).
• Prior to methodology validation and in order to optimize the protein extraction technique same methodologies were compared with a variation adapted from Oppen-Berntsen et al. (1990). Another extraction solution was compared as shown in Phase 3 (Table 1), which contained only 8 M urea and a protease inhibitor cocktail (Table 2) [14], which would allow not performing the protein precipitation step required with the SDS solution. However, the highest protein concentration was still obtained with the SDS (74.28 ± 13.65 µg/mL) extraction solution (Fig. 3), which was significantly higher (*p* < 0.001) than the BL (23.37 ± 3.31 µg/mL) and U8M (35.92 ± 3 µg/mL) solution.

• The methods chosen for Phase 2 were statistically compared by *t*-test using RStudio software version 4.2.2. Normality and homogeneity were confirmed. Three replicates in duplicate were performed for each treatment. Was found that the SDS treatment (76.44 ± 26.39 µg/mL) resulted in a significantly higher protein concentration (*p* = 0.01259) than the BL treatment (46.31 ± 5.16 µg/mL) (Fig. 2).

**Fig. 3:**
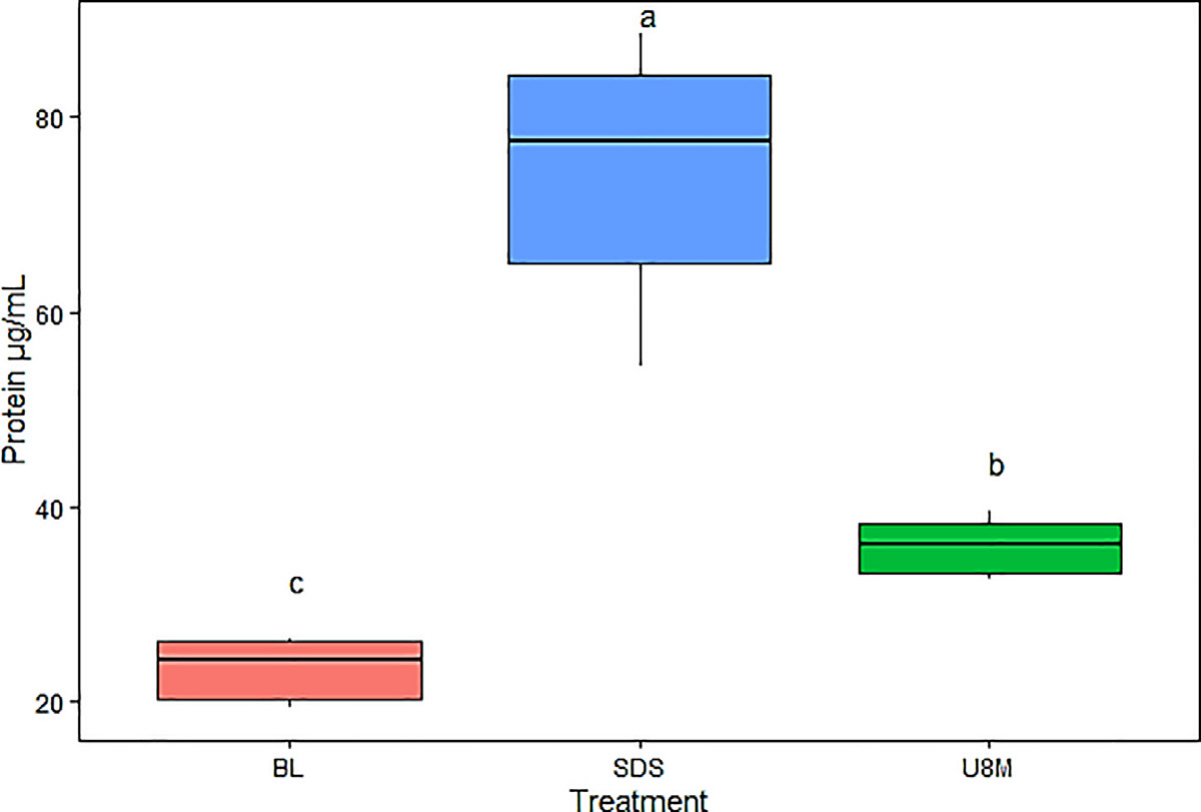
Protein concentration obtained in Phase 3 validation of the methodology for protein extraction from the chorion of Atlantic salmon (*Salmo salar*) embryos at the pigmented eye stage (280 ATU). Logarithmic transformation of the data was performed. Normality and homogeneity were checked by Shapiro-Wilk and Levene´s test, respectively. With a significance level of 95%, was found a highly significant difference (*p* < 0.001) between BL (23.37 ± 3.31 µg/mL), SDS (74.28 ± 13.65 µg/mL) and U8M (35.92 ± 3 µg/mL). Different letters indicate a significant difference between treatments. SDS: Tris–HCl 100 mM, Urea 8 M, 1% SDS, β-mercaptoethanol 300 mM and EGTA 10 Mm and 1% protease inhibitor cocktail; BL: Buffer lysis protein extraction solution (Tris 50 mM, NaCl 100 mM, EDTA 1 mM, EGTA 2.5 mM, Tween-20 0.1% and PMSF 100 µg/mL). U8M: Urea 8 M and 1% protease inhibitor cocktail.

## Method validation

• Finally, the validation phase (Table 1), is detailed below from washing the samples to running the SDS-Page gel. As a next step it is expected to perform Western Blot of *S. salar* embryo chorion proteins to identify the specific proteins, not only at the 280 ATU stage, but at various stages of development (e.g. before activation/hydration/fertilization of the oocytes, after fertilization and/or at hatching).

### Washing of chorion samples

**Fig. 4:**
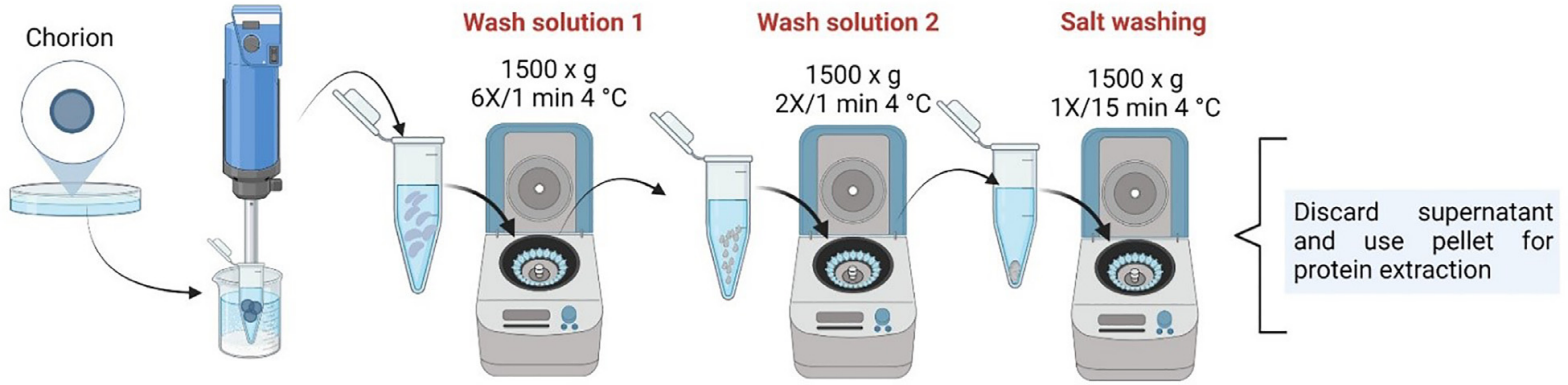
Procedure for washing chorion samples from Atlantic salmon (*Salmo salar*) embryos. Wash solution 1: EDTA 100 mM, NaCl 500 mM and protease inhibitor 1 µL/100 mL; wash solution 2: NaCl 500 mM and protease inhibitor 1 µL/100 mL.

In order to remove the presence of possible bacteria and/or fungi, debris, excess yolk and embryo remains, looking only for the presence of proteins belonging to the chorion during its extraction, a series of washes were carried out using the solutions previously used with Atlantic cod (*Gadus morhua*) for washing this casing [15]. The washing procedure (Fig. 4) was carried out as shown below:

• Obtaining the chorion of *S. salar* embryos in washing solution 1 (Table 2) and remove the excess yolk.
• The chorion samples immersed in clean wash solution 1, were placed in an Eppendorf or Falcon tube, depending on the amount of sample, and cut with a digital dispenser (IKA™ ULTRA-TURRAX™ T 18 Digital Disperser) for 30 to 60 s at a speed of 7400 rpm, continuously moving the sample towards and away from the dispenser, to achieve smaller pieces of chorion. The tubes were always kept inside a container containing ice to avoid heating of the sample and denaturation of the proteins (Supplementary Fig. 2).
• The samples were then centrifuged at a speed of 1500 x g for 1 min, six times, with the same washing solution, always discarding the supernatant.

Two more washes were performed with solution 2 (Table 2) at 1500 x g for 1 min with a final wash with H_2_Od containing protease inhibitor cocktail (1 µL/mL) to remove excess salts (1500 x g for 15 min).

### Extraction of chorion proteins

**Fig. 5:**
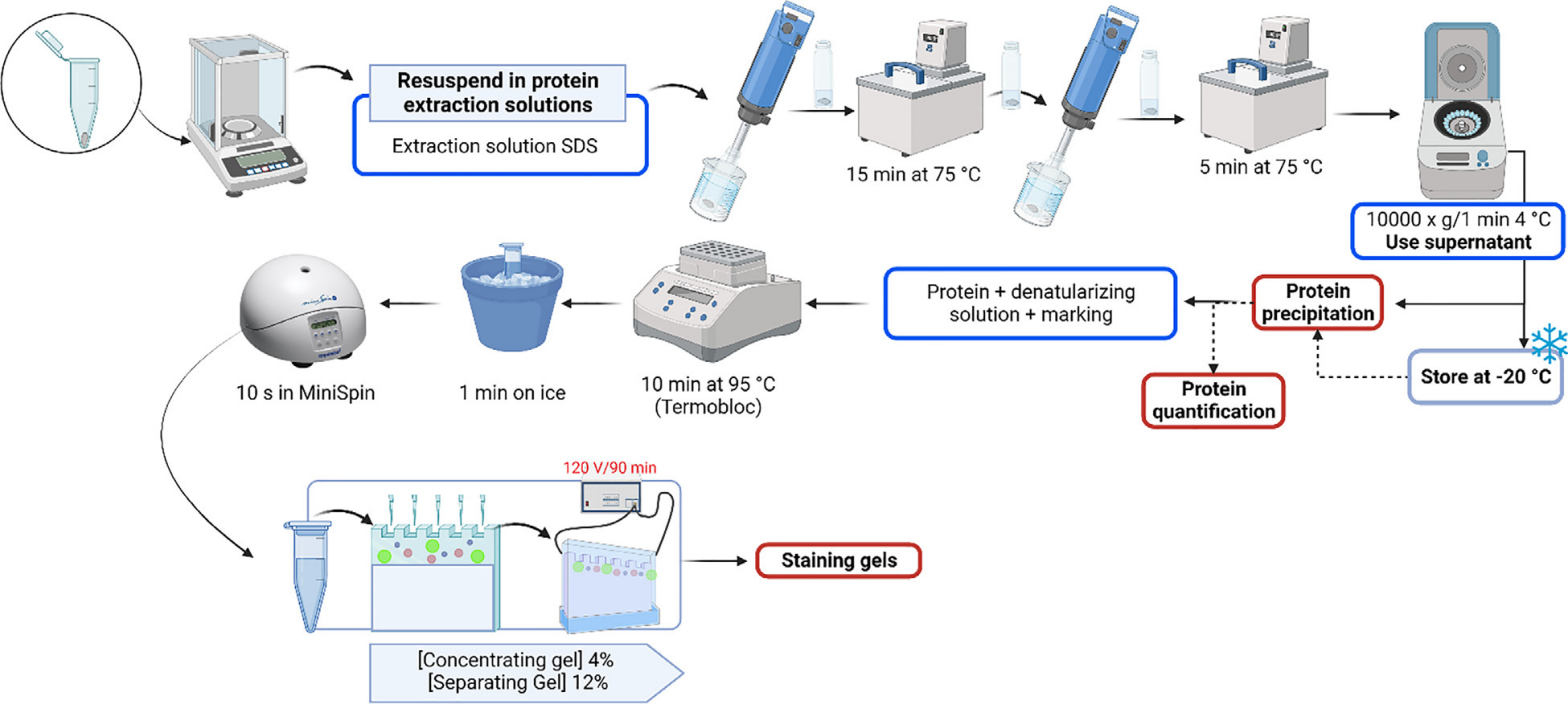
Extraction process of proteins from the chorion of Atlantic salmon (*Salmo salar*) embryos and at the pigmented eye stage (280 UTA) using the methodology of chemical extraction with SDS solution (Tris–HCl 100 mM, Urea 8 M, 1% SDS, β-mercaptoethanol 300 mM and EGTA 10 Mm and 1% protease inhibitor cocktail) and mechanical (digital dispenser, IKA™ ULTRA-TURRAX™ T 18 Digital Disperser) extraction. Samples were stored at −20 °C when precipitation, quantification and/or gel running were not performed continuously. The figure also summarizes the process of preparing the denaturation of the samples and running the SDS-PAGE gel.

**Fig. 6:**
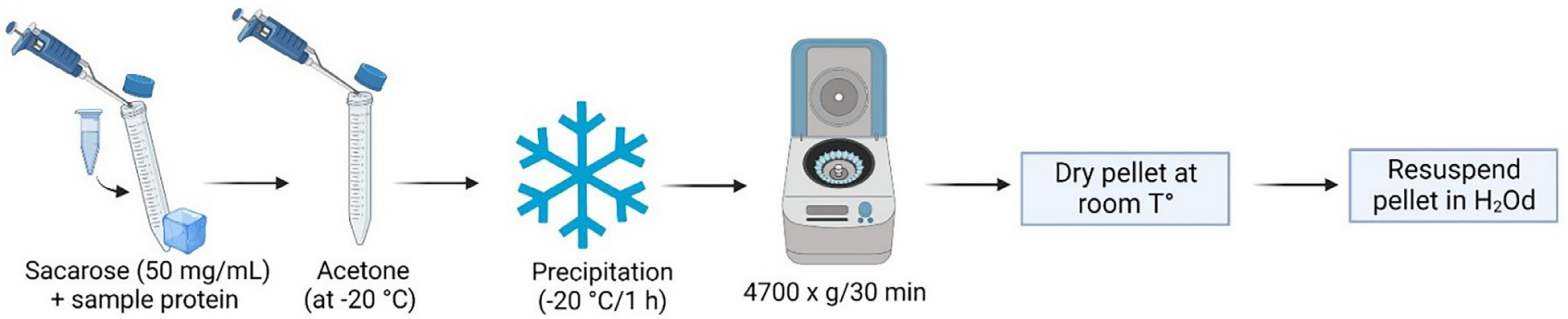
Protein precipitation from the chorion of Atlantic salmon (*Salmo salar*) embryos at the pigmented eye stage (280 ATU).
• The protein quantification was carried out by the Bradford method.
• The extracted proteins were stored at −20 °C until electrophoresis, which is explained later.

After washing the chorion samples, protein extraction (Fig. 5) was performed as shown below:

• Discard H_2_Od supernatant from the last wash step. A 0.05–0.08 g sample was weighed (in triplicate) in a Pico Glass Vial of 7 mL and 1 mL of SDS extraction solution was added.
• Place the samples in a water bath at 75 °C for 15 min and then homogenize each sample with the digital dispenser for 20 s as indicated above, washing its disperser between each sample.
• Return the samples to the water bath for 5 min and then centrifuged at 10,000 x g for 1 min at 4 °C (clarification) after homogenizing each sample again.
• Transfer the supernatant to an Eppendorf tube. Samples were stored at −20 °C.
• Protein precipitation (Fig. 6) for the supernatant of the samples extracted with the SDS solution was performed.

### Protein precipitation

• The sample volume obtained after protein extraction was transferred to a 15 mL Falcon tube and 50 mg/mL sucrose were diluted. The samples were always kept on ice.
• Add four acetone (at −20 °C) volumes to the samples. Place at −20 °C for 1 h for precipitation.
• Centrifuge at 4700 x g for 30 min at 4 °C.
• Discard supernatant and air-dry the pellet (Pellet formed in Supplementary Fig. 3).
• Resuspend the pellet in 100 µL of H_2_Od.

### Protein quantification

• *Bradford* was performed according to manufacturer's instructions (BIO-RAD Cat #500–0006). Briefly, a 10 mg/mL BSA solution was prepared to create the calibration curve.
• The spectrophotometer multimodal microplates (SynergyTM HT) was set at 595 nm and readings were made in a 96-well plate.
• A blank with H_2_Od was considered. A 200 µL volume of Bradford solution was added to each well. Measurements were performed in triplicate.
• Each of the samples from the different treatments containing the proteins extracted from the chorion were measured in triplicate. In addition to precipitation, another alternative for the quantification of protein concentration was applied. Thus, the samples, after protein extraction, were diluted in H_2_Od (dilution factor of 10) in order to decrease the SDS concentration to 0.1%, avoiding its interference with this colorimetric method. The same way the protein was diluted was also done with the calibration curve.
• The protein concentration of the samples was calculated from the linear regression equation of the calibration curve.

### Preparation of the gels

The reagents and concentrations used for gel preparation and loading are listed in Table 2.

• Mount the previously cleaned glasses with ethanol.
• Prepare the separating gel by adding the PSA and TEMED solutions last. Add the gel up to about 1 to 2 cm under the short glass.
• To even out the surface of the gel add a few drops of isopropanol and remove it once the separating gel is polymerized dried with absorbent paper.
• Prepare the concentrating gel by adding finally the PSA and TEMED solutions and pour to the overflow. Finally, place the comb (15 well, 1 mm, 26 µL) and let it polymerize.

### Sample preparation and loading

• After precipitation and resuspending the samples in H_2_Od an aliquot of the sample (**3** µg of protein) was taken and mixed with Protein loading buffer (5X concentrated) to load a final maximum volume of 20 µL per well.
• Before loading into the wells each sample was denatured at 95 °C in a Block Heater for 10 min and finally on ice for 1 min.
• Each sample was centrifuged in MiniSpin for 10 s and finally loaded on the gel (maximum 20 µL). Each replicate was loaded in triplicate for validation.
• Remove the comb after polymerizing the concentrate gel and install the system in the chamber.
• Load the samples in the wells available in the gel concentrator.
• Add the 1X running buffer between the space of the glasses and outside the glasses up to the maximum fill line.
• Load 3 µL of the pre-stained protein ladder in the first well and 20 µL of the samples in the following wells.
• Connect on the chamber at 120 V for 90 min.
• Stain the gel with Coomassie blue (according to the manufacturer's instructions), diluted as shown in Table 2. Staining was carried out for 30 min in a Rocker Shaker. After this time, each gel was washed with H_2_Od to remove excess Comassie blue and the distaining solution (Table 2) was added and left overnight. After this time the gel was washed with abundant H_2_Od and photographed.

## Conclusions and future considerations

**Fig. 7:**
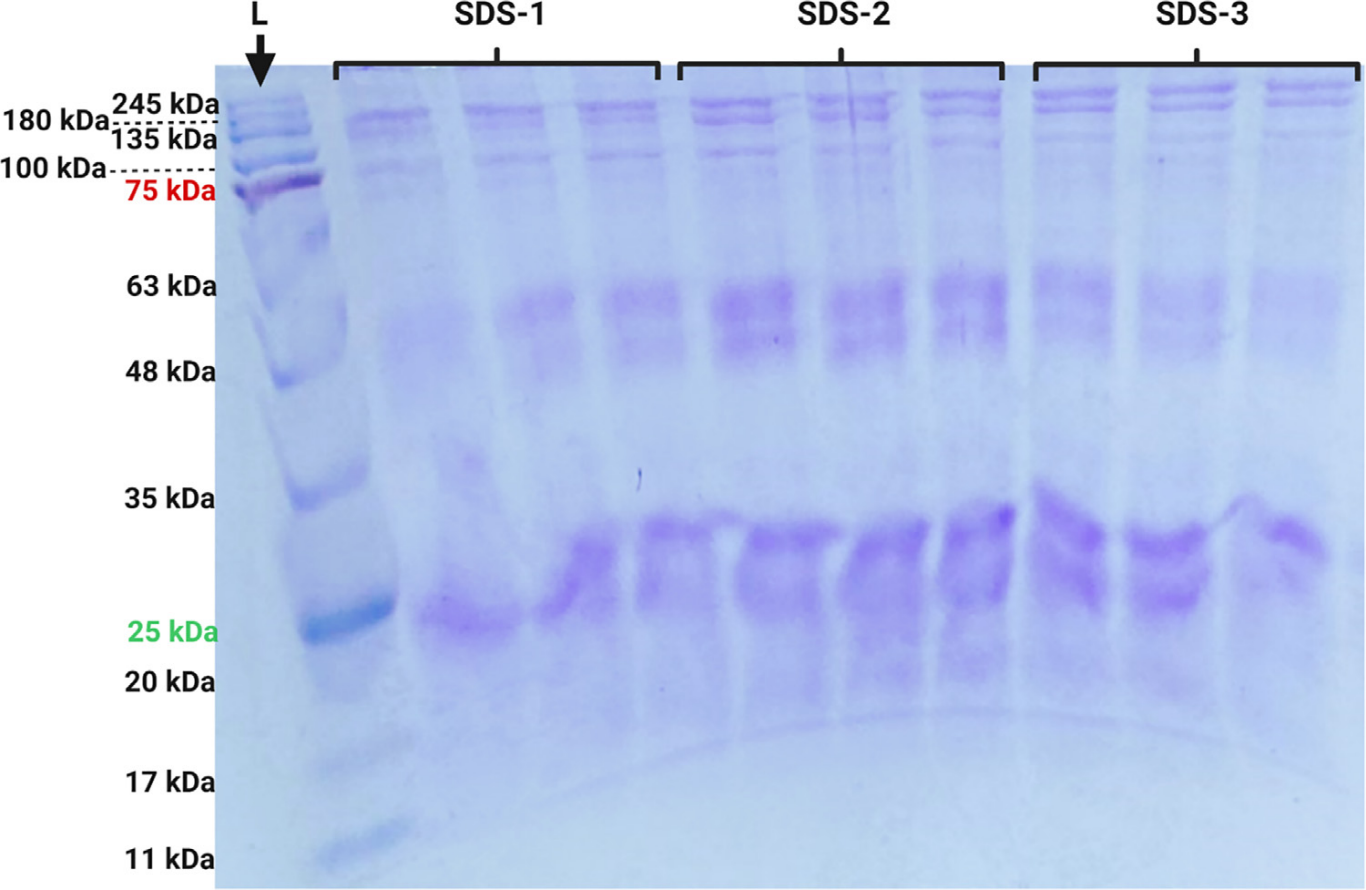
Electrophoresis with SDS-polyacrylamide gel after chorion protein extraction from Atlantic salmon (*Salmo salar*) embryos at 280 ATU. Three µg of proteins were loaded per well. Pre-stained protein ladder (L) and replicates (SDS-1, 2 and 3) with 3 repetitions.
• This protocol will serve as a starting point for the identification of the protein profile and future characterization of the different cases of chorion malformations identified in Atlantic salmon embryos and the possible differences between them.
• This standardized chorion-specific protein detection protocol is a valuable tool in the early identification of gamete and embryo quality in fish. Methodology that optimizes extraction times and concentration of chorion proteins, being a faster and more efficient protocol. Furthermore, it uses smaller volumes of biological samples and chemical reagents (reduces economic expenses), this last point being very important in terms of waste management in the laboratory and reducing its impact on the environment.

• For the extraction of proteins from the chorion, β-mercaptoethanol, SDS are required in order to obtain the highest amount of proteins present in the chorion samples of *S. salar* embryos at 280 ATU.
• Considering the reagents present in the extraction solution, methods such as protein precipitation or dilution are necessary. With the experience obtained throughout the standardization of this method, it is recommended to dilute (dilution factor of 10) the protein samples before evaluating the concentration using the *Bradford* protocol. However, other alternatives will continue to be tested in order to find a method that makes the measurement of protein concentration more efficient.
• Different from what was found by Jaramillo et al. [9] who found a total of four bands after gel staining in electrophoresis, in this case, in embryo chorion at 280 ATU more than 4 bands were stained, with approximate weights between 25 kDa and 180 kDa (Fig. 7). This may be due to the stage of embryonic development of the Atlantic salmon chorion. It happens that at the moment of hatching, the coriolysin (hatching enzyme) degrades the innermost layer of the chorion to facilitate its rupture by the embryo, for this reason, a necessary step to continue is to identify and to compare each of these proteins in the different stages of embryonic development.

## Ethics statements

The study was conducted in accordance with the Research Ethics Committee of the Universidad Católica de Temuco (No 112,201/22).

## Fundings

This research was funded by 10.13039/501100002850FONDECYT-REGULAR No 1211246; 10.13039/501100010751FONDECYT-INICIACIÓN No 11230690.

## CRediT authorship contribution statement

**Maritza Pérez-Atehortúa:** Conceptualization, Methodology, Software, Validation, Formal analysis, Investigation, Data curation, Writing – original draft, Writing – review & editing, Visualization. **Stefania E. Short:** Conceptualization, Methodology, Validation, Formal analysis, Investigation, Data curation, Writing – original draft, Writing – review & editing, Visualization, Supervision. **Cristian Aranzaez-Rios:** Conceptualization, Methodology, Software, Validation, Formal analysis, Investigation, Data curation, Writing – original draft, Writing – review & editing, Visualization. **Jorge Farías:** Resources, Supervision, Project administration, Funding acquisition. **Ricardo Pinheiro S. Oliveira:** Writing – review & editing, Visualization. **Wellison Amorim Pereira:** Writing – review & editing, Visualization. **Jennie Risopatrón:** Investigation, Writing – review & editing, Visualization. **Iván Valdebenito:** Conceptualization, Resources, Data curation, Project administration, Funding acquisition. **Elías Figueroa Villalobos:** Conceptualization, Methodology, Software, Validation, Formal analysis, Investigation, Data curation, Writing – original draft, Writing – review & editing, Visualization, Supervision.

## Declaration of Competing Interest

The authors declare that they have no known competing financial interests or personal relationships that could have appeared to influence the work reported in this paper.

## Data Availability

No data was used for the research described in the article.

## Appendix Supplementary materials

Image, application 1
