# Supplementary material for: Preparation and extraction of chorion proteins from Salmo salar embryos at the pigmented eye stage for electrophoresis with SDS-polyacrylamide gel
Source: MethodsX. 2023 Dec 23;12:102533. doi: 10.1016/j.mex.2023.102533 (PMC10784690; doi:10.1016/j.mex.2023.102533)
Supplement: Supplementary file 1 [file mmc1.docx]

**Supplementary material**

**Table S1.** Protein extraction methodologies from the chorion of different fish species.

| **Species** | **Solubization of chorion protein** | | **Authors** |
| --- | --- | --- | --- |
|  | **Mechanical extraction** | **Chemical extraction** |  |
| Goldfish (*Carassius auratus*) | Dounce tissue grinder in an ice bath was used with 10 strokes. This was followed by protein solubilization with ovarian tissue to purify the chorion samples. | Chorion was solubilized in sample buffer (0.01 M k buffer (pH 7), 1% SDS, 0.1 M dithiothreitol (DTT), 10% glycerol, 0.001% bromophenol blue) | (Cotelli et al., 1988) |
| Atlantic cod (*Gadus morhua*) | 30 - 60 s sonication using Branson B-15 cell disruptor. Applied after adding the chemical extraction solution. | Buffer B: 100 mM Tris/HCl (pH 8.0), containing urea 8 M, 1% SDS, 300 mM mercaptoethanol and 10 mM of EGTA. Incubated twice in water bath at 70°C (15 min and 5 min) | (Oppen-Berntsen et al., 1990) |
| Rainbow trout (*Oncorhynchus mykiss*) | Chorion samples were homogenised with several strokes with Dounce tissue grinder in an ice bath. Applied during chorion purification. | TNE (50 mM Tris HC1, 125 mM NaC1,lO mM EDTA, pH 7.2) containing 0.1% Triton X-100 and 1 mM PMSF | (Brivio et al., 1991) |
| Zebrafish (*Danio rerio*) | Chorion samples were homogenised with several strokes with Dounce tissue grinder in an ice bath. Applied after adding the chemical extraction solution. | Tris/HCl 0.0625 M (pH 6.8), 2% SDS, DTT 100 mM, glycerol 10%, bromophenol blue 0.05% | (Bonsignorio et al., 1996) |
| Gilthead seabream (*Sparus aurata*) | Microcentrifuge tube homogenizer and Sonicator. Applied after adding the chemical extraction solution. | Buffer A: 50 mM Tris/HCl (pH 8.0) and urea 8 M | (Modig et al., 2008) |
|  |  | Buffer B: 100 mMTris/HCl, pH 9.5, 1 M guanidium chloride and 2% β-mercaptoethanol |  |
| Atlantic salmon (*Salmo salar*) | Cutting of the chorion with a scalpel, followed by maceration in a mortar. Applied prior to chemical extraction | Urea 8 M in 0.1 M sodium phosphate buffer, pH 6.8. Incubated for 15 min at 100°C | (Jaramillo et al., 2012) |
| Common carp (*Cyprinus carpio*) and grass carp (*Ctenopharyngodon idellus*) | Before adding the extraction chemical solution, a powder was obtained using liquid nitrogen. The samples were sonicated (ultrasonic processor three times on ice after adding the extraction solution. | Lysis buffer (Urea 8 M, 1% Protease Inhibitor Cocktail) | (Wang et al., 2022) |

**References of supplementary material**

Bonsignorio, D., Perego, L., Del Giacco, L., & Cotelli, F. (1996). Structure and macromolecular composition of the zebrafish egg chorion. *Zygote (Cambridge, England)*, *4*(2), 101–108. https://doi.org/10.1017/S0967199400002975

Brivio, M. F., Bassi, R., & Cotelli, F. (1991). Identification and characterization of the major components of the Oncorhynchus mykiss Egg Chorion. *Molecular Reproduction and Development*, *28*(1), 85–93. https://doi.org/10.1002/MRD.1080280114

Cotelli, F., Andronico, F., Brivio, M., & Lamia, C. L. (1988). Structure and composition of the fish egg chorion (Carassius auratus). *Journal of Ultrastructura and Molecular Structure Research*, *99*, 70–80. https://doi.org/doi.org/10.1016/0889-1605(88)90034-1

Jaramillo, R., Goicoechea, O., Garrido, O., & Molinari, E. (2012). Caracterización electroforética de las proteínas del corion normal y del corion duro de Salmo salar. *Archivos de Medicina Veterinaria*, *44*(1), 59–65. https://doi.org/10.4067/S0301-732X2012000100009

Modig, C., Raldúa, D., Cerdà, J., & Olsson, P. E. (2008). Analysis of vitelline envelope synthesis and composition during early oocyte development in gilthead seabream (Sparus aurata). *Molecular Reproduction and Development*, *75*(8), 1351–1360. https://doi.org/10.1002/mrd.20876

Oppen-Berntsen, D. O., Helvik, J. V., & Walther, B. T. (1990). The major structural proteins of cod (Gadus morhua) eggshells and protein crosslinking during teleost egg hardening. *Developmental Biology*, *137*(2), 258–265. https://doi.org/10.1016/0012-1606(90)90252-E

Wang, Y., Chen, F., He, J., Chen, J., Xue, G., Zhao, Y., Peng, Y., & Xie, P. (2022). Comparative ultrastructure and proteomics of two economic species (common carp and grass carp) egg envelope. *Aquaculture*, *546*(July 2021), 737276. https://doi.org/10.1016/j.aquaculture.2021.737276


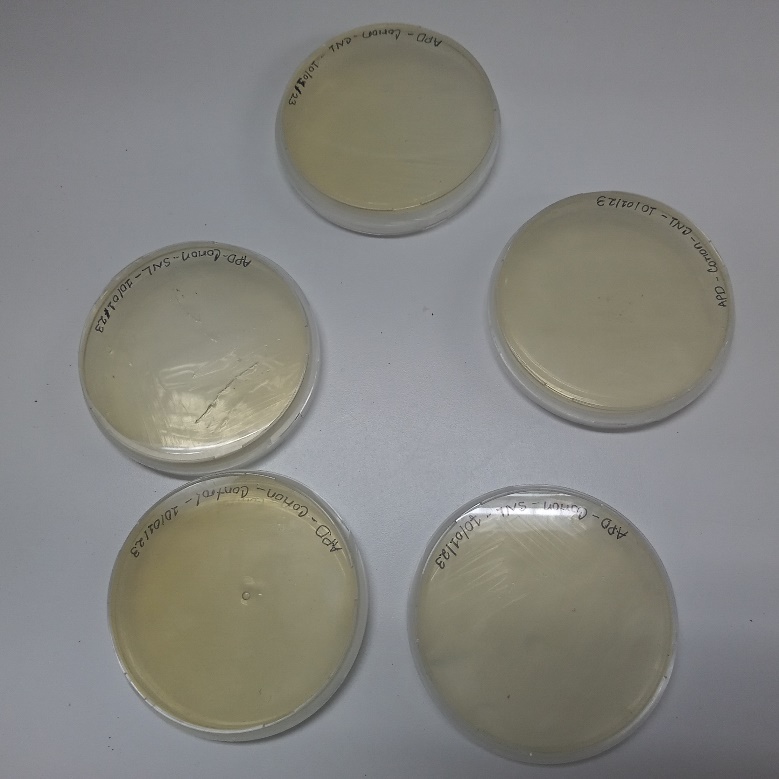

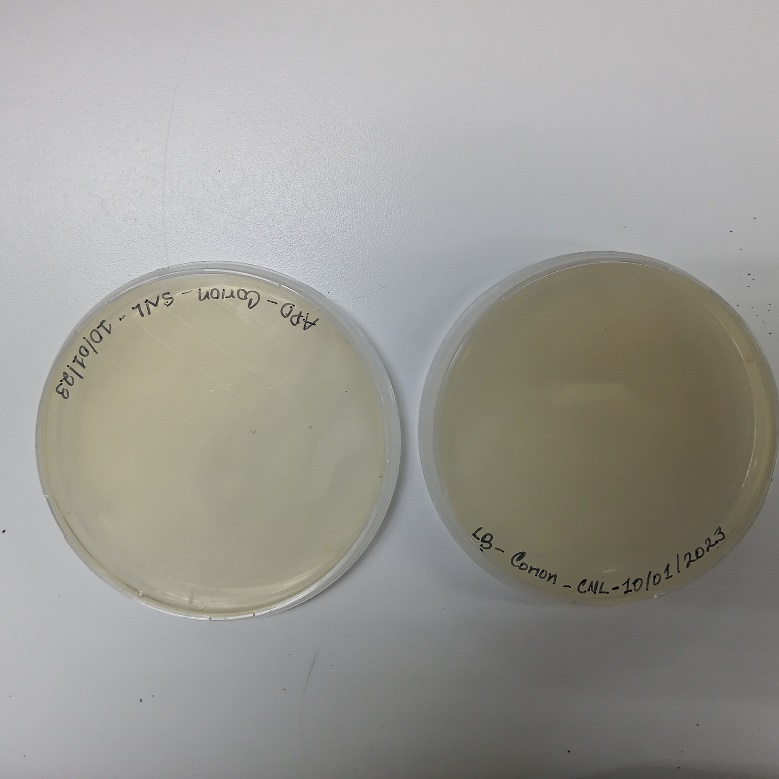


**Figure S1.** Fungal culture in Potato Dextrose Agar Acid (PDA) medium and bacteria culture in Luria-Bertani (LB) medium seven days after washing of chorion samples from Atlantic salmon (Salmo salar) embryos at pigmented eye stage (280 ATU), with and without the use of liquid nitrogen between washes. The images show that there was no growth of these microorganisms during the observation period.

**Table S2.** First phase of protocol standardization for protein extraction from the chorion of Atlantic salmon (Salmo salar) embryos at the pigmented eye stage (280 ATU).

| **Validation phase 1** | | | | | | |
| --- | --- | --- | --- | --- | --- | --- |
| **Chorion washing** | **Protein extraction** | | **Precipitation** | **[protein] µg/mL** | **Sample mass (g)** | **Ratio*** |
|  | **Chemical** | **Mechanical** |  |  |  |  |
| Liquid nitrogen | SDS | Sonicator | Y | 26.57 | 0.074 | 0.359 |
|  | SDS | Homogenizer | Y | 70.10 | 0.059 | 1.180 |
|  | SDS | Mortar | Y | 43.11 | 0.080 | 0.540 |
|  | SDS | Liquid nitrogen | Y | 37.88 | 0.075 | 0.506 |
|  | BL | Sonicator | N | 63.90 | 0.130 | 0.493 |
|  | BL | Homogenizer | N | 59.40 | 0.118 | 0.506 |
|  | BL | Mortar | N | 66.70 | 0.103 | 0.649 |
|  | BL | Liquid nitrogen | N | 62.81 | 0.063 | 0.997 |
| Without liquid nitrogen | SDS | Sonicator | Y | 75.36 | 0.105 | 0.715 |
|  | SDS | Homogenizer | Y | 77.60 | 0.097 | 0.797 |
|  | SDS | Mortar | Y | 133.18 | 0.064 | 2.073 |
|  | SDS | Liquid nitrogen | Y | 119.89 | 0.058 | 2.060 |
|  | BL | Sonicator | N | 57.21 | 0.119 | 0.482 |
|  | BL | Homogenizer | N | 49.62 | 0.087 | 0.572 |
|  | BL | Mortar | N | 55.84 | 0.042 | 1.346 |
|  | BL | Liquid nitrogen | N | 30.96 | 0.067 | 0.461 |

*The ratio was calculated by dividing the protein concentration obtained ([protein] µg/mL) by the sample weight (µg) of chorion used for extraction. The two methods with the highest ratio were chosen for a Phase 2 of protocol standardization. Precipitation was only applied to treatments in which SDS extraction solution was used, due to the interference of 1% SDS with the Bradford technique for quantifying protein concentration. Y: protein precipitation was performed; N: protein precipitation was not performed.

**C**

**B**

**A**


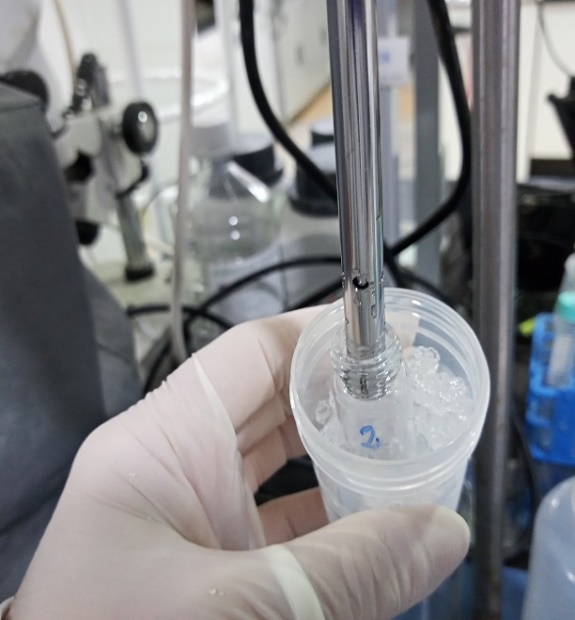

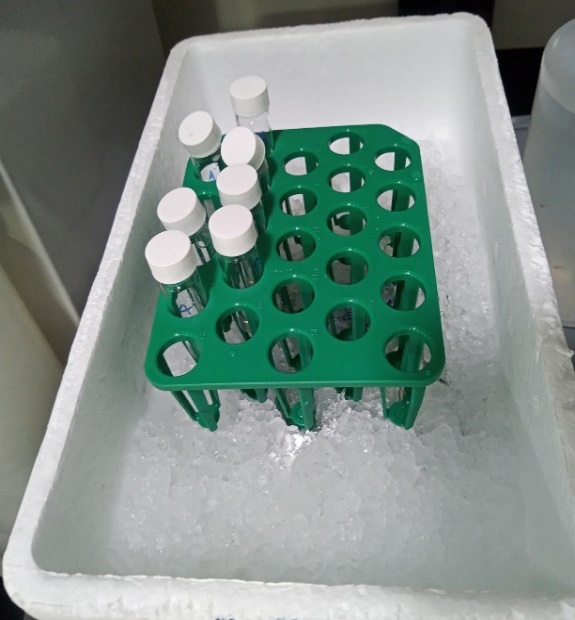

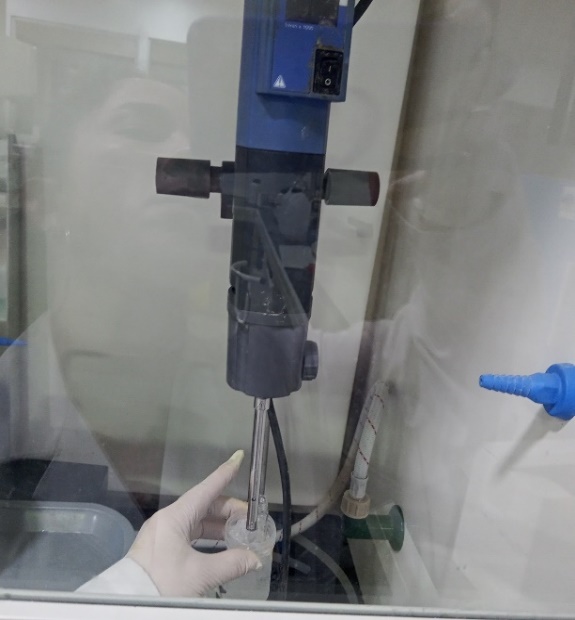

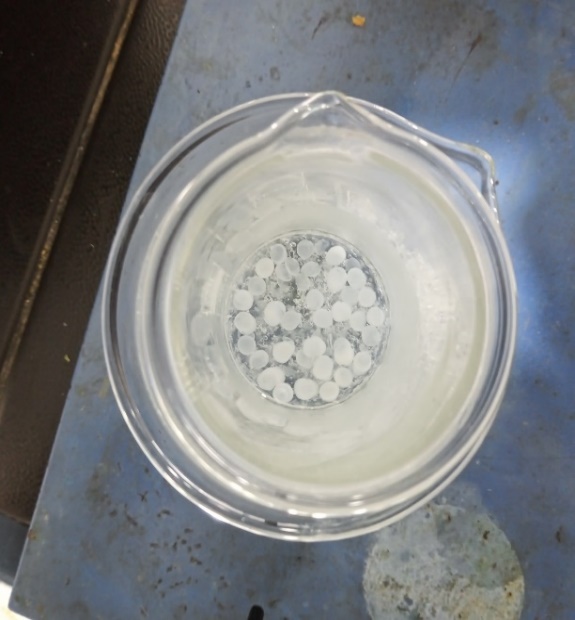

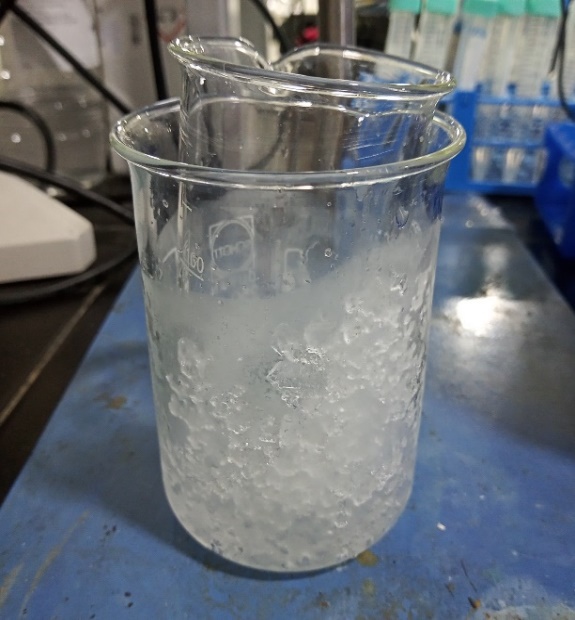

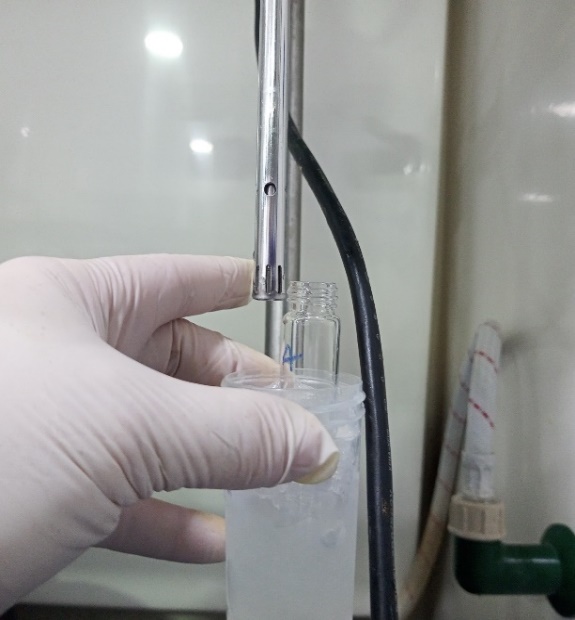


**Figure S2.** Handling of chorion samples from Atlantic salmon (Salmo salar) embryos at the pigmented eye stage (280 ATU) during the process of cutting the samples before washing (A) and protein extraction with a homogenizer digital dispenser (IKA™ ULTRA-TURRAX™ T 18 Digital Disperser) (B and C).


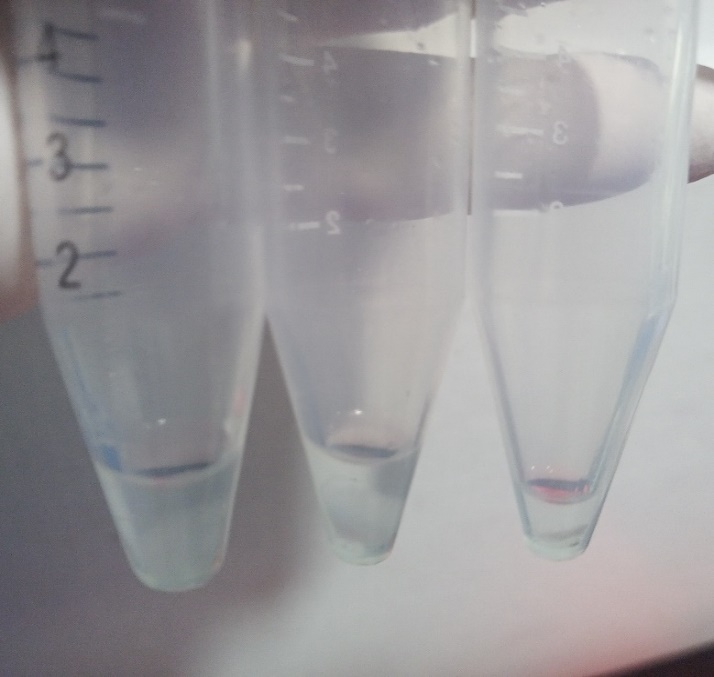

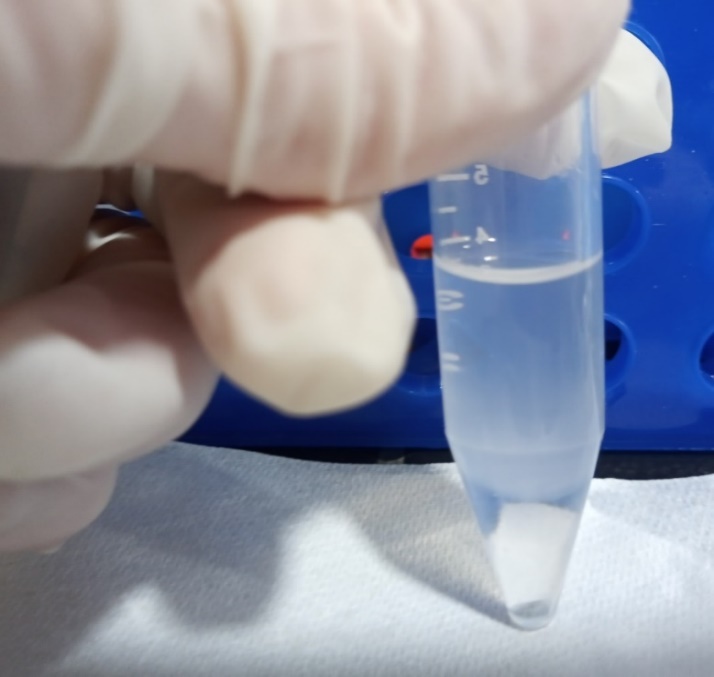


**Figure S3.** Pellet formed (arrows) after protein precipitation with sucrose and acetone.
